# Andrographolide Inhibits Ovariectomy-Induced Bone Loss via the Suppression of RANKL Signaling Pathways

**DOI:** 10.3390/ijms161126039

**Published:** 2015-11-17

**Authors:** Tao Wang, Qian Liu, Lin Zhou, Jin Bo Yuan, Xixi Lin, Rong Zeng, Xiaonan Liang, Jinmin Zhao, Jiake Xu

**Affiliations:** 1 Research Centre for Regenerative Medicine, Guangxi Medical University, Guangxi 530021, China; liulianwang@hotmail.com (T.W.); luoboqian@hotmail.com (Q.L.); sisic_082531@hotmail.com (X.L.); 15877171979@163.com (R.Z.); zhaojingmin@126.com (J.Z.); 2 Department of Orthopedics Surgery, the First Affiliated Hospital of Guangxi Medical University, Guangxi 530021, China; lxn120@126.com; 3 Guangxi Key Laboratory of Regenerative Medicine, Guangxi Medical University, Guangxi 530021, China; 4 School of Pathology and Laboratory Medicine, the University of Western Australia, Perth WA 6009, Australia; 20463692@student.uwa.edu.au (L.Z.); jinbo.yuan@research.uwa.edu.au (J.B.Y.)

**Keywords:** andrographolide, osteoclastogenesis, RANKL, OVX, bone loss

## Abstract

Osteoporosis is a debilitating skeletal disorder with an increased risk of low-energy fracture, which commonly occurs among postmenopausal women. Andrographolide (AP), a natural product isolated from Andrographis paniculata, has been found to have anti-inflammatory, anti-cancer, anti-asthmatic, and neuro-protective properties. However, its therapeutic effect on osteoporosis is unknown. In this study, an ovariectomy (OVX) mouse model was used to evaluate the therapeutic effects of AP on post-menopausal osteoporosis by using micro-computed tomography (micro-CT). Bone marrow-derived osteoclast culture was used to examine the inhibitory effect of AP on osteoclastogenesis. Real time PCR was employed to examine the effect of AP on the expression of osteoclast marker genes. The activities of transcriptional factors NF-κB and NFATc1 were evaluated using a luciferase reporter assay, and the IκBα protein level was analyzed by Western blot. We found that OVX mice treated with AP have greater bone volume (BV/TV), trabecular thickness (Tb.Th), and trabecular number (Tb.N) compared to vehicle-treated OVX mice. AP inhibited RANKL-induced osteoclastogenesis, the expression of osteoclast marker genes including cathepsin K (Ctsk), TRACP (Acp5), and NFATc1, as well as the transcriptional activities of NF-κB and NFATc1. In conclusion, our results suggest that AP inhibits estrogen deficiency-induced bone loss in mice via the suppression of RANKL-induced osteoclastogensis and NF-κB and NFATc1 activities and, thus, might have therapeutic potential for osteoporosis.

## 1. Introduction

The maintenance of bone homeostasis involves two coordinated actions; namely bone formation (by osteoblasts) and bone resorption (by osteoclasts). The balance between the activities of osteoblasts and osteoclasts is critical to ensure normal bone structure. When this balance is disrupted, an increase or a decrease in bone mass will occur [1]. Enhanced osteoclast formation and/or activation are known mechanisms underlying osteolytic diseases such as osteoporosis, Paget’s disease of bone, metastatic bone disease, and erosive arthritis [2]. Thus, osteoclasts have become key targets in the treatment of osteoporosis and other osteoclast-related osteolytic diseases [3].

Osteoclasts are specialized giant polykaryons, which are formed by the fusion of mononuclear progenitors of monocyte/macrophage lineage, and are responsible for bone resorption. Osteoclast formation, survival, and activation are regulated by several key cytokines. Although macrophage colony-stimulating factor-1 (MCSF-1) is necessary for the proliferation and survival of osteoclast progenitor cells [4], the receptor activator of nuclear factor-κB (NF-κB) ligand (RANKL) is a critical cytokine for osteoclast differentiation [5]. Upon binding to its receptor RANK on osteoclast progenitor cells, RANKL recruits an adaptor molecule, tumor necrosis factor (TNF) receptor-associated factor 6 (TRAF6). This in turn results in a cascade of intracellular signaling, which includes IκB kinase (IKK), RANKL-Induced Nuclear Factor-κB (NF-κB), extracellular signal-regulated kinases (ERK), c-Jun N-terminal kinase (JNK), p38 MAPK (P38 mitogen-activated protein kinases), Akt, Proto-oncogene tyrosine-protein kinase Src (c-Src), activator protein 1 (AP-1) and nuclear factor and activator of transcription (NFATc1) [6,7]. Among these signaling molecules, NF-κB plays an indispensable role in osteoclastogenesis. Following the phosphorylation of IκBα by the activated IκB kinase, NF-κB is released from the NF-κB/IκBα complex, and translocated to the nucleus, where it binds to its DNA target sequences, and triggers the expression of specific genes important for the formation of osteoclasts [8]. Based on the current understanding of this molecular mechanism, the inhibition of RANKL signaling by anti-RANKL antibodies or anti-NF-κB compounds is of prime focus in developing anti-catabolic therapies for osteoporosis.

In recent years, the use of natural herbal products to inhibit osteoclast formation and osteoclast-related diseases has garnered attention. Andrographolide (AP), a bicyclic diterpenoid lactone, is a naturally occurring product isolated from the leaves of a traditional Chinese herb, Andrographis paniculata, and is used for the treatment of respiratory infections, inflammation, fever, and diarrhea [9]. Modern pharmacological studies suggest that AP exerts anti-cancer [10], anti-inflammatory [11,12], anti-asthmatic [13], and neuro-protective effects [14] partly by regulating NF-κB. Furthermore, AP has the potential to prevent inflammatory osteolysis [15,16] and human breast cancer-induced bone loss [17]. However, the therapeutic effect of AP on post-menopausal osteoporosis is unknown. Thus, in the present study, we investigated the direct effects of AP on postmenopausal osteoporosis using an ovariectomy (OVX) mouse model, and explored its molecular mechanisms of action.

## 2. Results

### 2.1. Andrographolide (AP) Inhibits Ovariectomy-Induced Bone Loss in Vivo

**Figure 1:**
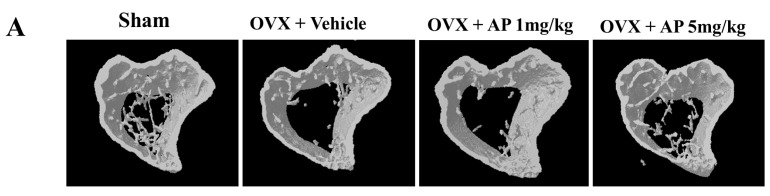

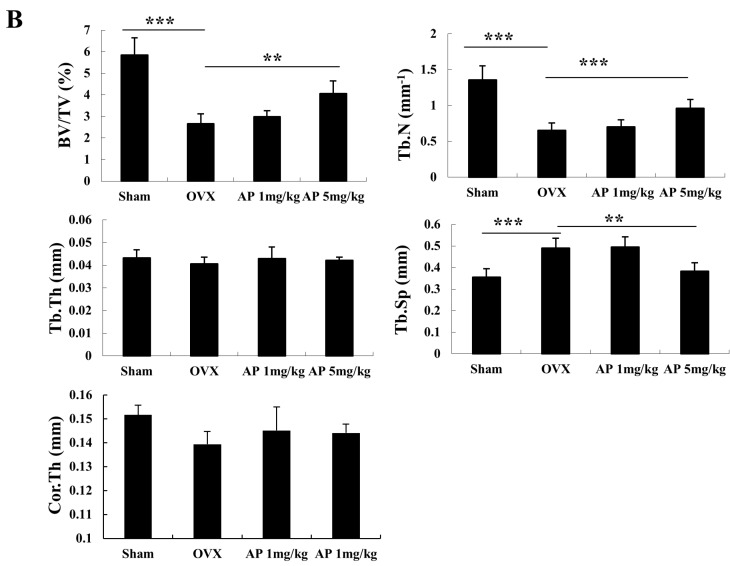
Andrographolide inhibits OVX-induced bone loss. A week after ovariectomy, mice were injected with vehicle (1% DMSO) or AP (1 or 5 mg/kg) every two days for a six-week period. (**A**) representative 3D images showing tibias of mice scanned with micro-CT; and (**B**) analyses of microstructural indices including bone volume per tissue volume (BV/TV), trabecular number (Tb.N), trabecular thickness (Tb.Th), trabecular separation (Tb.Sp), and cortex thickness (Cor.Th) calculated by micro-CT. *n* = 6, ** *p* < 0.005; *** *p* < 0.001.

To examine the effects of AP on bone loss *in vivo*, OVX mice were used to mimic postmenopausal osteoporosis. Tibias from OVX mice treated with AP or vehicle were analyzed using micro-CT. The trabecular bone mass in OVX mice was markedly reduced compared to sham-operated mice at seven weeks post-ovariectomy, which was confirmed by 3D reconstruction (Figure 1A) and quantitative bone parameters (Figure 1B). AP treated OVX group showed a dose-dependent therapeutic effect (at dose of 5 mg/kg/2 days) in preventing bone loss induced by OVX, with significant increases in bone volume per tissue volume (BV/TV) and trabecular number (Tb.N), and a decrease in trabecular separation (Tb.Sp) as compared to vehicle treated OVX group (Figure 1). AP had no significant effect on cortex thickness (Cor.Th) (Figure 1B).

### 2.2. AP Inhibits Receptor Activator of Nuclear Factor-κB Ligand (RANKL)-Induced Osteoclastogenesis

**Figure 2:**
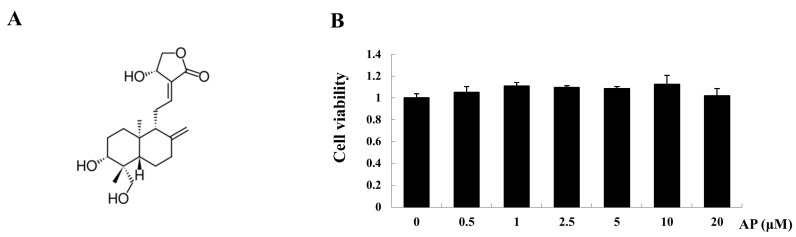

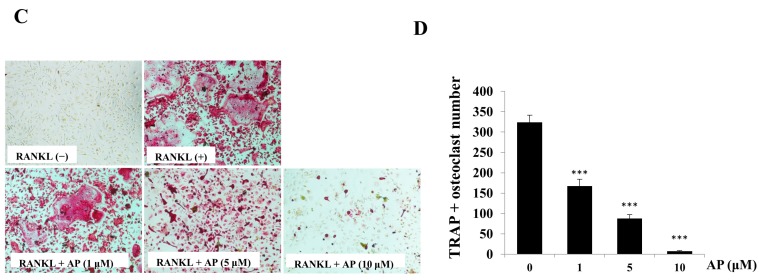
Effect of andrographolide on RANKL-induced osteoclast formation. BMMs were cultured in the presence of RANKL with different doses of AP for five days. (**A**) Chemical structure of AP; (**B**) cell viability measured using a MTS assay, in which BMMs were treated with varying concentrations of AP for 48 h; (**C**) Light microscopy images showing the effect of AP (one, five, and 10 μM) on RANKL-induced osteoclast formation (Mag = 20×); and (**D**) cell count of osteoclast like cells with TRACP-positive multinucleated cells. *** *p* < 0.001.

To examine the effect of AP on osteoclastogenesis, we first tested the effect of AP on bone marrow macrophages (BMM) cell viability using a MTS assay. As shown in Figure 2B, AP had no cytotoxic effects within the dose range of up to 20 μM. To determine the dose dependent effect of AP on RANKL-induced osteoclastogenesis, BMMs were cultured in the presence of RANKL with varying concentrations of AP, and stained for tartrate-resistant acid phosphatase (TRACP) activity. After five days of RANKL stimulation, the BMMs were fully differentiated into osteoclasts (Figure 2C). However, when AP was added to BMMs during the course of osteoclastogenesis, osteoclast formation was inhibited in a dose-dependent manner, as demonstrated by the significantly decreased total number of osteoclasts. Addition of AP at the dose of one, five, and 10 μM resulted in a huge reduction in osteoclast number (Figure 2D). Taken together, these data showed a dose-dependent inhibitory effect of AP on RANKL-induced osteoclastogenesis.

### 2.3. AP Suppresses Osteoclast-Related Gene Expression

**Figure 3:**
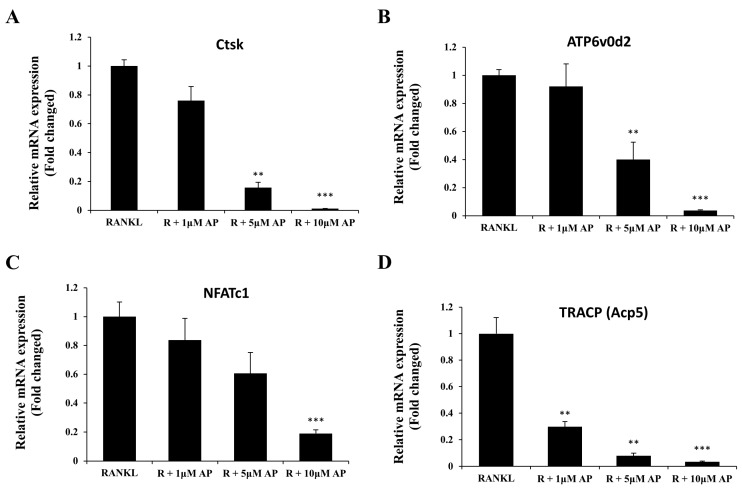
Andrographolide attenuates RANKL-induced gene expression. mRNA analysis of Ctsk (**A**); ATP6v0d2 (**B**); NFATc1(**C**) and TRACP (**D**) genes from BMMs that were treated with M-CSF (25 ng/mL), RANKL (50 ng/mL) and varying doses of AP (1, 5 and 10 μM) for five days. mRNA level were determined by real-time PCR and normalized to gene expression of GAPDH. All experiments were run in triplicate. ** *p* < 0.005; *** *p* < 0.001.

To further investigate the effect of AP on osteoclast differentiation, the expression of osteoclast-related genes, which are up-regulated in response to RANKL, were examined by real-time PCR. As demonstrated in Figure 3, the expressions of osteoclasts marker genes cathepsin K (Ctsk), ATP6v0d2, NFATc1, and TRACP (Acp5), were induced by RANKL. Treatment with AP at the concentration of 10 μM, decreased the RANKL-induced mRNA expression of these genes in a dose-dependent manner. This result is consistent with its inhibitory effect on osteoclast formation as shown in Figure 2.

### 2.4. AP Suppresses RANKL-Induced Nuclear Factor-κB (NF-κB) Activation

**Figure 4:**
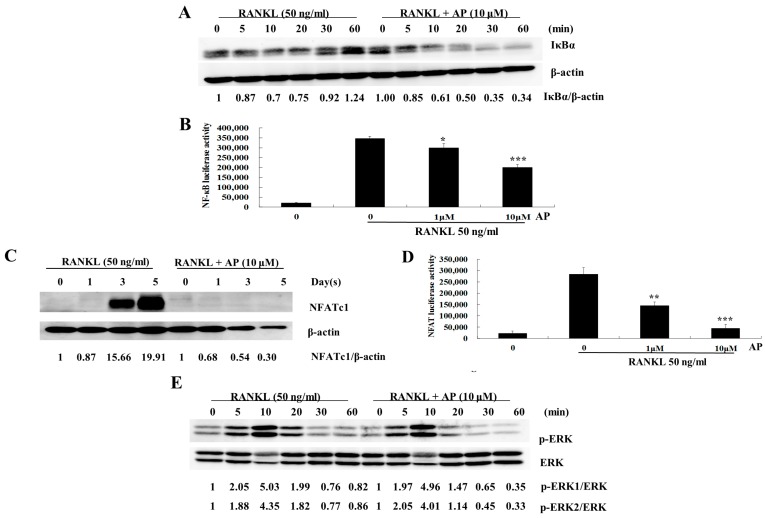
Andrographolide inhibits RANKL-induced NF-κB and NFATc1 activities. (**A**) BMM were seeded at a density of 5 × 10^5^ per well. After attachment overnight, cells were pre-treated with AP for 1 h and then stimulated with M-CSF (25 ng/mL) and RANKL (50 ng/mL) for indicated times. Lysate of cells were collected by using RIPA Lysis Buffer, and separated with 10% SDS-PAGE following by transferring onto nitrocellulose membrane. The membrane was blocked and probed with antibodies to IκBα and β-actin. Signal intensities of bands were detected, and shown as a ratio of IκBα/β-actin; (**B**) RAW 264.7 cells, which transfected with 3κB-Luc-SV40 reporter gene, were pre-treated with one or 10 μM AP for 1 h followed by RANKL stimulation for 6 h. Cell lysates were collected and examined for luciferase activity; (**C**) BMMs were treated with AP (10 μM) and stimulated with and M-CSF and RANKL for indicated times. Cell lysates were collected for Western blot analysis using antibodies to NFATc1 and β-actin; (**D**) RAW 264.7 cells stably transfected with an NFATc1 luciferase reporter construct were cultured with AP at one or 10 μM in the presence of RANKL. Luciferase activity was measured after 24 h; and (**E**) BMMs were treated with AP (10 μM), and stimulated with RANKL (50 ng/mL). Cell lysates were analyzed by Western blot with antibodies to ERK and p-ERK. All experiments were run in triplicate. Student’s t-test was used for statistical analysis. * *p* < 0.05; ** *p* < 0.005; *** *p* < 0.001.

The NF-κB pathway has been shown to play an essential role in RANKL-induced osteoclastogenesis [18]. To explore the possible mechanism underlying the inhibitory effect of AP on osteoclastogenesis, we investigated the effect of AP on IκBα degradation and NF-κB activation. As shown in Figure 4A, IκBα degradation was observed at 10 min after RANKL stimulation, and then IκBα was re-synthesised at 30 min (Figure 4B). AP (10 μM) treatment delayed the RANKL-induced IκBα degradation, which was most evident at 60 min. The effect of AP on modulating NF-κB transcriptional activity was also tested using RAW 264.7 cells stably transfected with an NF-κB luciferase reporter construct (3κB-Luc-SV40). As shown in Figure 4B, NF-κB transcriptional activation increased significantly after RANKL stimulation compared to the control group. However, treatment with AP showed a dose-dependent suppression of the NF-κB activation at concentrations of one and 10 μM.

### 2.5. AP Suppresses RANKL-Induced Nuclear Factor of Activated T-Cells (NFATc1) Activation

NFATc1, a transcription factor belonging to the NFAT family, regulates the expression of osteoclast specific genes, such as Ctsk, TRACP (Acp5), calcitonin receptor (Ctr), and osteoclast-associated receptor (Oscar) [19,20]. As stated above, AP inhibited RANKL-induced gene expression of Ctsk, TRACP (Acp5), and NFATc1. Therefore, we further investigated its regulatory effect on the protein level of NFATc1. Western blot analysis showed that the protein level of NFATc1 was increased at day three after RANKL stimulation, and was more pronounced at day five (Figure 4C). Notably, when AP (10 μM) was added to the BMM culture, the protein level of NFATc1 was suppressed. The transcriptional activity of NFATc1 was also analyzed by using a luciferase reporter gene assay. The transcriptional activity of NFAT was dramatically up-regulated by RANKL, but this effect was strongly inhibited by AP in a dose dependent manner (Figure 4D).

### 2.6. AP Does not Affect RANKL-Induced Extracellular Signal-Regulated Kinases (ERK) Phosphorylation

ERK is one of the three subfamilies of MAPKs, which also play an important role in RANKL-induced osteoclastogenesis [21,22]. Therefore, we explored whether this signaling was intervened by AP during RANKL stimulation. The results showed that AP has no obvious effect on the ERK phosphorylation induced by RANKL (Figure 4E).

## 3. Discussion

Osteoporosis is the most common bone disorder, caused by excessive bone resorption by osteoclasts without adequate bone formation by osteoblasts [23]. Therefore, osteoclasts become the key targets of commonly-used drugs in the treatment of osteoporosis and other osteoclast-related osteolytic iseases [24]. Many plant-derived polyphenols, such as mangiferin [25], curcumin [26], and naringin [27] have been shown to attenuate osteoporosis through negative regulation of osteoclastogenesis. In this study, we demonstrated that AP effectively inhibits bone loss induced by OVX. We also found that AP was capable of attenuating osteoclastogenesis via the inhibition of RANKL-induced NF-κB and NFATc1 activities. Understanding the therapeutic effect of AP on bone loss induced by estrogen deficiency and the underlining mechanisms might provide vital information for developing potential therapies against post-menopausal osteoporosis and osteoclast-related diseases.

Increased bone resorption, trabecular thinning, and decreased connections between the remaining trabeculae are the predominant features of post-menopausal estrogen withdrawal [28], and ovariectomy could mimic this phenomenon in animals. In this study, we evaluated whether AP can prevent bone loss induced by estrogen deficiency in C57BL/6 mice. The data showed that OVX mice lead to damaged bone microstructure and decreased trabecular bone compared to sham-operated mice. Intraperitoneal injection of AP significantly suppressed the bone loss induced by OVX, accompanied by increased BV/TV and Tb.N, and decreased Tb.Sp (Figure 1). Our results also suggested that AP had no significant effect on Cor.Th. However, the direct effects of AP on osteoblasts and bone formation require further investigation.

Osteoclasts, originated from monocyte-macrophage lineage, are unique cells in body responsible for bone resorption [29]. Excessive osteoclast formation and activity cause bone disorders, such as osteoporosis, metastatic bone disease, and inflammatory arthritis, where bone resorption exceeds bone formation [30]. In this study, we demonstrated that AP inhibits osteoclast formation in a dose-dependent manner. In order to choose an optimal dose of AP for BMMs culture, cell viability was examined at varying concentrations of AP. The result showed that AP has no cytotoxic effect on BMMs within the dose range of up to 20 μM. In the experiment of cell culture, AP at the dose of five or 10 μM, inhibited the formation of osteoclasts and the expression of osteoclast maker genes, including Ctsk, ATP6v0d2, NFATc1, and TRACP. These results are consistent with the therapeutic effect of AP on bone loss *in vivo*.

Upon RANKL binding, RANK recruits an adaptor protein TRAF6, which then induces a cascade of intracellular events. Among the molecules involved, NF-κB and NFATc1 are two well-documented transcription factors that are important for osteoclastogenesis [31,32]. The stimulatory signal from TRAF6 triggers phosphorylation and subsequent degradation of IκBα, leading to the activation of NF-κB. It is well known that the activation of NF-κB is critical for osteoclast differentiation, resorption, and survival [33]. Notably, NF-κB p50^−/−^ and p52^−/−^ double-knockout mice exhibit severe osteopetrosis, since they fail to generate mature osteoclasts [31].

Previous reports have identified the inhibitory ability of AP on NF-κB activity. For example, in 293 cells, AP covalently modified NF-κB and abrogated the TNF-α-induced NF-κB binding to its DNA target sequence [34]. In the permanent cerebral ischemia rat model, AP exerted neuro-protective effects by suppressing NF-κB activation [14]. The attenuation of RANKL-induced NF-κB activity by AP in osteoclasts is consistent with these results and might be part of the underlining mechanisms for the inhibitory effect of AP on osteoclastogenesis *in vitro* and bone loss *in vivo*.

NFATc1, a well-known master transcription factor, regulates the expression of osteoclast-specific genes such as the Ctsk, TRACP (Acp5), MMP9, Ctr, ATP6V0d2, and c-Src that are associated with osteoclast differentiation and function [19,35]. In addition, this function is regulated by NF-κB activity [32]. Conditional knockout of NFATc1 in mice resulted in a severe osteopetrotic phenotype and inhibition of osteoclastogenesis [32,36]. Although the exact details of the mechanism are unclear, here, we demonstrated that AP down-regulates RANKL-induced NFAT transcription in a dose-dependent manner. The protein level of RANKL-induced NFATc1 in osteoclasts was also suppressed significantly by AP. These results indicate that AP exerts the inhibitory effect on osteoclastogenesis partly by suppressing the NFATc1-mediated pathway. Therefore, AP not only affects the activation of NF-κB and NFATc1, but also the expression of their downstream genes, which contributes to its inhibitory effect on osteoclast formation and function.

Estrogen suppresses osteoclast formation by inhibiting osteoclastogenic cytokines such as IL-1, IL-6, IL-11, and TNF-α [37,38]. There is evidence that AP could also alleviate lipopolysaccharide (LPS)-induced osteolysis in mice [16]. Given that AP has strong anti-inflammatory effects and that inflammatory cytokine production positively influences osteoclastic bone resorption, it is possible that the therapeutic effect of AP on OVX mice bone mass is partly due to its anti-inflammatory property. In addition, there is a possibility of an estrogen-like effect of andrographolide that might contribute to its protective effect of OVX-induced bone loss. However, to our knowledge, there is no report that AP has an estrogen-like effect to date.

Taken together, our results indicate that AP inhibits osteoclastogenesis *in vitro* and bone loss in the OVX mouse model. Furthermore, our data demonstrate that the inhibitory effect of AP on osteoclasts is due to, at least in part, the suppression of RANKL-induced NF-κB and NFATc1 signaling pathways. Thus, AP might serve as a potential therapeutic agent for the treatment of osteoporosis and other osteoclast-related diseases.

## 4. Materials and Methods

### 4.1. Animals

Seven-week-old female C57BL/6 mice were purchased from the Academy of Military Medical Sciences, and housed in the SPF experimental center of Guangxi Medical University. The experimental procedures and animal facility were approved by the Animal Care and Welfare Committee of Guangxi Medical University (SYXK2009-0004). After a week of acclimatization, all animals were anaesthetized with 10% chloral hydrate and bilateral ovariectomy was performed, except for mice in the sham operated group (mice in the sham operated group were anaesthetized, and the ovaries were exposed but not removed). After post-surgery recovery for one week, all OVX mice were randomly divided into three groups (*n* = 6): OVX control group, AP 1 mg/kg group, and AP 5 mg/kg group. The AP groups were intraperitoneally injected with AP at corresponding concentrations in 1% DMSO in physiological saline, while the OVX control group received 1% DMSO as vehicles. All injections were administered every two days for a period of six weeks. At the end of treatment, all mice were sacrificed for the extraction of tibias, which were fixed in 4% paraformaldehyde (PFA) at room temperature (25 °C) for 24 h followed by three rinses with 1× PBS. The bone microarchitecture of proximal tibias was measured using a micro-tomography scanner (Skyscan, Aartselaar, Belgium) with the setting as following: 50 kV, 500 µA, constant exposure, 0.5 mm Al filter, voxel size 9 nm, 0.4° angular rotation. 3D structures were reconstructed using NRecon software (Skyscan). A region 0.5 mm from the growth plate and 1 mm in height within the proximal tibia was analyzed by CTAn (Skyscan) software to evaluated trabecular structure parameters, including bone volume per tissue volume (BV/TV), trabecular number (Tb.N), trabecular thickness (Tb.Th), and trabecular separation (Tb.Sp). Cortex thickness (Cor.Th) was evaluated through the same way but using a region 5 mm from the growth plate.

### 4.2. Reagents and Antibodies

AP (99.50%, assay by HPLC) was purchased from Mansite (Chengdu, Sichuan, China). For *in vitro* study, AP was dissolved in DMSO and the final concentration of DMSO in the culture was less than 0.02%. Alpha Modified Eagle’s Medium (αMEM) and fetal bovine serum (FBS) were obtained from Gibco BRL (Gaithersburg, MD, USA). Primary antibodies for β-actin, IκBα and NFATc1 were purchased from Cell Signaling Technology (Danvers, MA, USA). Recombinant GST-RANKL proteins were expressed and purified as previously described [39].

### 4.3. In Vitro Osteoclastogenesis Assay

To evaluate the effect of AP on osteoclast differentiation, *in vitro* osteoclastogenesis assay was performed. BMMs were obtained from six-week-old C57BL/6 mice by flushing femurs and tibias with αMEM and were cultured in a T-75 flask with a complete medium containing αMEM and 25 ng/mL macrophage-colony stimulating factor (M-CSF). The medium was changed every two days. After 5–7 days of proliferation and attaining confluence, BMMs were harvested by washing with phosphate-buffered saline (PBS) and trypsinization for 30 min. To generate osteoclasts, BMMs were plated in 96-well plates at a density of 6 × 10^3^ cells per well, incubated overnight, and stimulated with M-CSF (25 ng/mL) and RANKL (50 ng/mL). Simultaneously, the cells were treated with or without AP. The culture medium was replaced every 2–3 days. After five days, the cells were fixed with 4% paraformaldehyde in PBS) for 10 min at room temperature, rinsed with 1× PBS, and stained with tartrate-resistant acid phosphatase (TRACP) staining buffer. TRACP-positive multinucleated cells with more than three nuclei were counted as osteoclast-like (OCL) cells.

### 4.4. Cytotoxicity Assay

BMMs were plated in 96-well plates (6 × 10^3^ cells per well) and cultured overnight at 37 °C. The cells were then treated with M-CSF (25 ng/mL) and varying concentrations (0, 0.5, 1, 1.25, 5, 10, 20 μM) of AP. After culturing for two days, 50 µL MTS was added to each well and incubated for 4 h. The optical density (OD) was measured at 490 nm wavelength by using a BMG plate reader (BMG, Offenburg, Germany).

### 4.5. Real Time Polymerase Chain Reaction (Real-Time PCR)

For real-time PCR, BMMs were seeded into 6-well plate at a density of 1 × 10^5^ cells per well and cultured with M-CSF (25 ng/mL) and RANKL (50 ng/mL) for five days in the presence of varying AP concentration (1, 5 and 10 μM). Total RNA was isolated using the RNAeasy Mini Kit (Qiagen, Valencia, CA, USA) and reverse-transcription was conducted using 1 μg of total RNA with oligo-dT primers at 42 °C for 1 h. All PCRs were performed using 2 µg of respective cDNA. Quantification of mRNA was performed using ABI 7500 Sequencing Detection System (Applied Biosysterms, Foster City, CA, USA) with SYBR Premix Ex Tag kit (TaKaRa Biotechnology, Dalian, Liaoning, China), and normalized to housekeeping gene GAPDH. The following cycling parameters were used: 40 cycles of denaturation at 95 °C for 15 s and annealing at 60 °C for 60 s. The mouse primers used in this study are presented in Table 1.

**Table 1:** List of primers used for RT-PCR.

mRNA	Primer	Sequences (5ʹ–3ʹ)	Product (bp)
Ctsk	Forward	GGGAGAAAAACCTGAAGC	350
	Reverse	ATTCTGGGGACTCAGAGC	
ATP6v0d2	Forward	GTGAGACCTTGGAAGACCTGAA	176
	Reverse	GAGAAATGTGCTCAGGGGCT	
NFATc1	Forward	CAACGCCCTGACCACCGATAG	392
	Reverse	GGCTGCCTTCCGTCTCATAGT	
TRACP (Acp5)	Forward	TGTGGCCATCTTTATGCT	462
	Reverse	GTCATTTCTTTGGGGCTT	
GAPDH	Forward	ACCACAGTCCATGCCATCAC	452
	Reverse	TCCACCACCCTGTTGCTGTA	

### 4.6. Western Blot Assay

BMMs were seeded into six-well plates at a density of 5 × 10^5^ cells per well and incubated overnight. Following pre-treatment with AP for 1 h, the cells were stimulated with RANKL (50 ng/mL) for the indicated times. The cells were then lysed with RIPA Lysis Buffer (Millipore, Billerica, MA, USA) containing a protease inhibitor cocktail (Roche, Basel, Switzerland). Proteins were separated by 10% SDS-PAGE, and then transferred onto a nitrocellulose membrane (Bio-Rad, Gladesville, NSW, Australia). Non-specific interactions were blocked using 5% skim milk powder (SMP) in 1× TBS-Tween (TBST) for one hour. After washing with 1× TBST, membrane was immunoblotted with antibodies against ERK, p-ERK, IκBα, NFATc1 and β-actin, visualized using enhanced chemiluminescence (ECL) reagents under a FujiFilm LAS-3000 system to detect the immunoreactivity. Signal intensities were quantified using NIH ImageJ software.

### 4.7. Luciferase Reporter Gene Assay for NF-κB and NFAT

To test the effect of AP on RANKL-induced NF-κB activation, RAW 264.7 cells stably transfected with a NF-κB luciferase construct (3κB-Luc-SV40) [40] were used in this assay. Cells were plated in 48-well plates and pre-treated with AP for 1 h, followed by RANKL (50 ng/mL) stimulation for 6 h. Cells were harvested and lysates isolated for luciferase assay. Luciferase activity was measured using a Promega Luciferase Assay system (Promega, Madison, WI, USA) according to manufacturer’s instructions. NFAT activity was test through the same way, but using RAW 264.7 cells which stably transfected with an NFATc1 luciferase reporter construct [41], and stimulated with RANKL for 24 h.

### 4.8. Statistical Analysis

All data are represented as mean ± standard deviation (SD) from three donors. Two-tailed Student’s t-test was used for statistical analysis. * *p* < 0.05, ** *p* < 0.01 or *** *p* < 0.001 were considered statistically significant.
